# Viruses and immunosenescence – more players in the game

**DOI:** 10.1186/s12979-019-0152-0

**Published:** 2019-06-24

**Authors:** Mikko Hurme

**Affiliations:** Faculty of Medicine and Health Technology, Tampere University, FI-33014 Tampere, Finland

**Keywords:** Immunosenescence, Virus infection, Endogenous retrovirus, Virome

## Abstract

Viral infections are common clinical problems in aged individuals often affecting both mortality and morbidity. The pathogenic mechanisms of the various viruses are not universal in aged individuals, i.e. the clinical disease may be caused by the reactivation of a virus which has stayed in the body in a latent form, or alternatively, the virus is exogenous, derived from the environment. However, it is now evident, that this concept is too simple. Recent data have shown that in our body, even in the blood of healthy individuals, there are large amount of various viruses, which seem to live in balance with our immune defense mechanism (viral normal flora?). Moreover, there is now data suggesting that remnants of ancient retroviral infections in our genome can be activated and show virus-like activities. The possible significance of these findings in immunosenescence is discussed.

## Introduction

Our body is in continuous contact with viruses and various defense mechanisms are used to prevent the entry or to eliminate the invader within the body. There is ample evidence demonstrating that the aging-associated decline of the immune system, i.e. immunosenescence, significantly weakens these mechanisms [1]. This is often observed in the case of common viral pathogens, e.g. influenzavirus [2]. On the other hand, it is known that at least some viruses may induce or modify immunosenescence and in this respect cytomegalovirus (CMV) is the classical and extensively investigated example [3]. However, there is now emerging evidence showing that the number of viruses or virus-like entities is much larger than expected i) next generation (NGS) RNA/DNA sequencing based approaches have shown that within our body there are large amounts of various viruses even without known clinical or biological significance (forming the virome) [4], i.e. the classical concept about the “sterility” of the inner body should be rejected ii) our genome contains mobile genetic elements (retrotransposons, endogeneous retroviruses (HERV), some of which may still be active and might modify the immune system [5]. In this review, these two concepts are briefly described and their role in immunosenescence is discussed.

### More viral candidates

The human body contains microbial communities (bacteria. fungi, viruses and protozoa) in its various compartments, often called the local microbiome (e.g. gut microbiome) and this can be further specified by the type of the microbe (e.g. gut virome). In traditional clinical microbiology changes in the composition of these microbiomes is analyzed in a simple and limited way, i.e. by searching known pathogenic microbes within these populations. However, during recent years the NGS –based analyses of all the DNA/RNA in the sample (i.e. metagenomic and metatranscriptomic analysis) and advances in bioinformatics have allowed the detection of all or at least most of the microbes in a given microbiome.

The results have been surprising. Changes in the microbiome (mainly gut bacteriome) have been observed to be associated with several environmental and life-style factors, aging, and with several disease states [6, 7]. These observations have been associative, i.e. the causal relationship e.g. with a given molecular activation pathway or with the pathogenic mechanisms in diseases have remained enigmatic. However, it is very likely that the effects of microbiome on human health starts already in childhood maybe having a role in the development and physiology of the body.

Analysis of the virome (including bacteriophages) is technically more challenging than that of the bacteriome. Viruses lack a clear sequence signatures (in contrast to bacteria) and therefore sequencing of large viral libraries is time-consuming and not easily applicable to a simultaneous analysis of large number of samples.

The first virome analyses have now been published [4]. It seems that the different compartments of the body harbor distinct viral communities. However, the total number of viruses is highly variable, 10^9^ particles per gram in the intestinal content, 10^7^/ml in the urine and 10^5^ /ml in the blood. Studies on gut virome have shown, that the most common viruses are not those infecting eukaryotic cells, but those infecting prokaryotic cells, bacteriophages, form a clear majority [8]. It has even been estimated that bacteriophages outnumber all forms of life on our planet [9].

The presence of phages was demonstrated about 100 years ago, and their life-cycle within the bacteria as well as their role in modulating the functions of bacteria, e.g. mediating toxin production or antibiotic resistance are all well known [10]. Moreover, based on their bactericidal effect, phages have been attractive candidates for anti-microbial therapy in humans.

This far the relationship between virome composition and immunosenescence is not known. However, there are several reports demonstrating changes in the gut virome compostion in diseases of immunological nature, e.g. type I diabetes [11]. Moreover, in immunocompromised patients (DOCK8 deficiency) the skin virome is clearly expanded [12]. Based on these, it could be expected that immunosenescence would have an influence on virome composition. However, its possible role in the aging-associated pathologies can presently only be speculated. Does the weaker immunity allow the presence of potentially pathogenic viruses in the blood of elderly individuals? It is also possible that this viral “normal flora” would have a protective effect, in analogy with the bacterial normal flora in several compartments of the human body.

An interesting question is also the role of bacteriophages. Aging is associated with changes in the gut bacteriome [13]. As bacteriophages are derived from the gut bacteria, it is probable that the phageome composition is also changed in aged individuals. It has been shown that some bacteriophages are able to induce the production of inflammatory cytokines in vitro [14]. As leakage of the gut is increased in aged individuals, it seems possible that this increased amount of proinflammatory bacteriophages or other viruses would contribute to inflammaging.

Finally, a common feature in these metagenomic analyses has been the high proportion of “dark matter”, i.e. sequences that do not align with the sequences of the reference human genome or those of known viruses or bacteria. It is probably at least partly caused by difficulties in the bioinformatics analyses, but it may also contain sequences of presently unknown viruses. Still more players in the field?

### Ancient invaders

About a half of the human genome is derived from mobile genomic elements. These elements can be categorized as transposons and retrotransposons, which differ in their mechanism of action. Transposons move by a “cut-and-paste” mechanism while retrotransposons use an RNA intermediate, i.e. a “copy-and-paste” mechanism. Retrotransposons can further be subdivided into long terminal repeat (LTR) and non-LTR elements. Human endogenous retroviruses (HERV) belong to the LTR subset. They comprise ca. 8% of the human genome, i.e. a significant proportion of our genome is derived from invasions by exogenous retroviruses during evolution [5, 15].

There is now increasing evidence that both the non-LTR and LTR retrotransposons are involved in the aging process. LINE-1, the main component of the non-LTR group (ca. 17% of the human genome), is the only active retrotransposon in the human body, and its insertional polymorphisms in the germline DNA are associated with several diseases, e.g. hemophilia [16]. This far there is no evidence showing associations between these genetic polymorphisms with the aging process or longevity in humans. However, as LINE-1 is continuously active it is possible that it induces somatic mutations during lifetime in this way modifying the aging process. The indications of the aging-associated increase in LINE-1 retrotransposonal activity are the increases in its copy number in the genome as well as in its RNA levels [17]. Although this LINE-1 –induced somatic retrotransposonal acivity is an attractive model, firm evidence of its functionality in the aging process is still missing. However, it is also possible that aging-associated increase in LINE-1 expression has an effect on aging associated inflammation via a different mechanism. De Cecco et al. [18] demonstrated that LINE-1 activity was clearly increased in the proinflammatory senescent associated secretory phenotype cells and activated the interferon –dependent classical anti-viral mechanism cGAS-STING, i.e. behaving like a virus.

As mentioned above, HERVs are relics of ancient retroviral infections in our genome comprising ca. 8% of our genome. They have lost their infective/retrotransposonal activity due to recombinations, deletions, and mutations during their millions years of history [5, 15]. HERVs have been shown to be involved in the pathogenesis of autoimmune diseases, activation of B cells and T regulatory cells and in malignant transformation [19], but the exact mechanisms of action are still elusive. Several of these studies are compromised by the fact that there are thousands of HERVs demonstrating different biological activities (or are totally inactive due to the lack of production of the HERV proteins, env, gag and pol). Also in this case the NGS RNA sequencing has provided a technical solution, i.e. allowing the determination of expression levels of all the individual proviruses if the genetic annotation data are available. Using this approach Nevalainen et al. [20] examined the expression of the proviruses in the HERV-K (HML-2) family. This family is the most recent entrant to our genome therefore containing several intact proviruses [21]. Out of the 91 proviruses circa one third was clearly expressed and this expression was significantly higher in elderly individuals in the case of only two proviruses (at 1q22 and at 10p14). It is unlikely that the weaker immune capacity would be responsible for these differences, but it is more probably due to the aging-associated decline of the epigenetic control [22]. The functional consequences of this increased expression are not yet clear. It is naturally possible that the retroviral DNA/RNA is recognized by the interferon-mediated anti-viral mechanisms in this way stimulating the inflammatory response, i.e. in a similar way as observed in the case of LINE-1. This would mean that the effect of HERVs is non-specific, depending simply on the total quantity of the DNA/RNA stimulus. However, it is obvious that the HERV encoded proteins (e.g. env) can be recognized by the immune system and such antibodies (autoantibodies?) can be detected in several disease states [5] but their functional significance is still largely unknown. There is one study showing that the titer of these antibodies is affected by aging, i.e. immunosenescence [23]. Moreover, already several years ago it was demonstrated that at least one HERV-K encoded env acts as a superantigen, i.e. causing a polyclonal activation of lymphocytes [24]. Maybe this superantigenic capacity, if it is a common character of several proviruses, is also involved in the exhaustion and deterioration of the immune system during aging.

## Conclusions

**Fig. 1:**
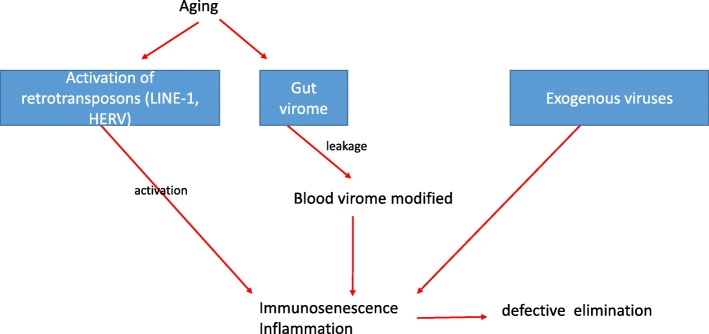
Viruses and immunosenescence – a tentativemodel

The data shown here indicate that the relationship between viruses, virosphere, and immunosenescence is more complex than previously thought. Firstly, the expressions of the repetitive elements in our genome (HERV, LINE-1) are able to activate immune/inflammatory responses in this way modulating the development of immunosenescence. Secondly, it is now evident that large amounts of viruses can be detected in our inner body, e.g. in the blood, which has previously thought to be sterile in healthy individuals. The origin of this virome, its pathogenic significance as well as the effect of immunosenescence on its composition are not yet known. The tentative role of these virus-mediated effects in immunosenescence is schematically shown in the figure below (Fig. 1).

## Data Availability

Not applicable (review).

## Abbreviations

CMV: Cytomegalovirus
HERV: Human endogenous retrovirus
LTR: Long terminal repeat
NGS: Next generation sequencing

## References

[1] Pawelec G (2012). Hallmarks of human “immunosenescence”: adaptation or dysregulation?. Immun Ageing.

[2] Weinberger B. Vaccines for the elderly: current use and future challenges. Immun Ageing. 2018;15(3). 10.1186/s12979-017-0107-2 PMC5778733 29387135

[3] Nicolich-Zugich J, Goodrum F, Knox K, Smithey MJ (2017). Known unknowns: how might the persistent herpesvirome shape immunity and aging. Curr Op Immunol.

[4] Zarate S, Taboada B, Yocupicio-Monroe M, Arais CF (2017). Human virome. Arch Med Res.

[5] Magiorkinis G, Belshaw R, Katzourakis A (2013). There and back again: revisiting the pathological roles of human endogenous retroviruses in the post-genomic era. Phil Trans B Soc B.

[6] Kho ZY, Lal SK (2018). The human gut microbiome – a potential control of wellness and disease. Front Microbiol.

[7] Cani PD (2018). Human gut microbiome: hopes, threats and promises. Gut.

[8] Chatterjee A, Duerkop BA (2018). Beyond bacteria: bacteriophage –eukaryotic host interactions reveal emerging paradigms of health and disease. Front Microbiol.

[9] Forde A, Hill C (2018). Phages of life – the path to pharma. Brit J Pharmacol.

[10] Sharma S, Chatterjee S, Datta S, Prasad R, Dubey D, Prasad RK, Vairale MG (2017). Bacteriophages and its applications: an overview. Folia Microbiol.

[11] Zhao G, Vatanen T, Droit L, Park A, Kostic AD, Poon TW, Vlamakis S, Siljander H, Härkönen T, Hämäläinen AM, Peet A, Tillman V, Ilonen J, Wang D, Knip M, Xavier RJ, Virgin HW (2017). Intestinal virome changes precede autoimmunity in type I diabetes-susceptible children. Proc Natl Acad Sci U S A.

[12] Tirosh O, Conlan S, Deming C, Lee-Lin SQ, Huang X, Comparative Sequencing Program NISC, Su HC, Freeman AF, Segre JA, Kong HH (2018). Expanded skin virome in DOCK8-deficient patients. Nat Med.

[13] Riaz Rajoka MS, Zhao O, Li N, Lu Y, Lian Z, Shao D, Shi J (2018). Origination, change, and modulation of geriatric disease-related gut microbiota during life. Appl Microbiol Biotechnol.

[14] van Belleghem JD, Clement F, Merabishvili M, Lavigne R, Vaneechoutte M (2017). Pro- and anti-inflammatory responses of peripheral blood mononuclear cells induced by Staphlycoccus aureus and Pseudomonas aeruginosa phages. Sci Rep.

[15] Roy-Engel AM (2012). LINEs, SINEs and other retroelements: do birds of a feather flock together?. Front Biosci.

[16] Hancks DC, Kazazian HH (2016). Roles for retrotransposon insertions in human disease. Mob DNA.

[17] Maxwell PH (2016). What might retrotransposons teach us about aging?. Curr Genetic.

[18] DeCecco M, Ito T, Petrashen AP, Elias AE, Skvir NJ, Criscione SW, Calgiana A, Brocculi G, Adney EM, Boeke JD, Le O, Beausejour C, Ambati J, Ambati K, Simon M, Seluanov A, Gorbunova V, Slagboom PE, Helfand SL, Neretti N, Sedivy JM (2019). L1 drives IFN in senescent cells and promotes age-associated inflammation. Nature.

[19] Katoh I, Kurata S (2013). Association of endogenous retroviruses and long terminal repeats with human disorders. Front Oncol.

[20] Nevalainen T, Autio A, Hamal Mishra B, Marttila S, Jylhä M, Hurme M (2018). Aging-associated patterns in the expression of human endogenous retroviruses. PLoS One.

[21] Subramanian RP, Wildschutte JH, Russo C, Coffin JM (2011). Identification, characterization, and comparative genomic distribution of the HERV-K (HML-2) group of human endogenous retroviruses. Retrovirology.

[22] Cardelli M (2018). The epigenetic alterations of endogenous retroelements in aging. Mech Ageing Dev.

[23] Kim HJ, Moon B, Lee JW, Kim SC, Kim H (2016). Age-related reduction of antibody response against the human endogenous retrovirus K envelope in women. Oncotarget.

[24] Sutkowski N, Conrad B, Thorley-Lawson DA, Huber BT (2001). Epstein-Barr virus transactivates the human endogenous retrovirus HERV-K18 that encodes a superantigen. Immunity.

